# Microbial Mechanistic Insight into the Role of Yeast−Derived Postbiotics in Improving Sow Reproductive Performance in Late Gestation and Lactation Sows

**DOI:** 10.3390/ani14010162

**Published:** 2024-01-04

**Authors:** Junlei Chang, Xinlin Jia, Yalei Liu, Xuemei Jiang, Lianqiang Che, Yan Lin, Yong Zhuo, Bin Feng, Zhengfeng Fang, Jian Li, Lun Hua, Jianping Wang, Zhihua Ren, De Wu, Shengyu Xu

**Affiliations:** 1 Key Laboratory for Animal Disease-Resistant Nutrition of China Ministry of Education, Ministry of Agriculture and Rural Affairs, Institute of Animal Nutrition, Sichuan Agricultural University, Chengdu 611130, China; junlei@stu.sicau.edu.cn (J.C.); 18483662636@163.com (X.J.); 2020214023@stu.sicau.edu.cn (Y.L.); 71310@sicau.edu.cn (X.J.); che.lianqiang@sicau.edu.cn (L.C.); linyan@sicau.edu.cn (Y.L.); zhuoyong@sicau.edu.cn (Y.Z.); fengb123d@163.com (B.F.); zfang@sicau.edu.cn (Z.F.); lijian522@hotmail.com (J.L.); hualun@sicau.edu.cn (L.H.); wangjianping@sicau.edu.cn (J.W.); wude@sicau.edu.cn (D.W.); 2 Sichuan Province Key Laboratory of Animal Disease and Human Health, Key Laboratory of Environmental Hazard and Human Health of Sichuan Province, College of Veterinary Medicine, Sichuan Agricultural University, Chengdu 611130, China; renzhihua@sicau.edu.cn

**Keywords:** yeast−derived postbiotic, sows, weaned piglets, fecal microorganism

## Abstract

**Simple Summary:**

Yeast−derived postbiotic (Y−dP) is a microbial product formed by sufficient anaerobic fermentation of yeast on a specific medium under specific process conditions. (Y−dP) plays an important role in improving feed utilization, preventing diseases, and enhancing body immunity. Our results showed that adding Y−dP to the diet of sows from late pregnancy to lactation can regulate the gut microbiota of sows during pregnancy, lactation, and piglets, improve the intestinal health of offspring piglets, and have a positive impact on improving sow reproductive performance. These findings provide theoretical reference for the application of (Y−dP) in sow production and are beneficial for reducing the use of antibiotics.

**Abstract:**

The purpose of this study is to investigate the effects of supplementing Yeast−derived postbiotics (Y−dP) to the diet of sows during late pregnancy and lactation on fecal microbiota and short−chain fatty acids (SCFA) in sows and their offspring weaned piglets, as well as the relationship between gut microbiota and SCFA, serum cytokines, and sow reproductive performance. A total of 150 sows were divided into three groups: control diet (CON), CON + Y−dP 1.25 g/kg, and CON + Y−dP 2 g/kg. The results showed that supplementing 0.125% Y−dP to the diet of sows can increase the content of isobutyric acid (IBA) in the feces of pregnant sows and reduce the content of butyric acid (BA) in the feces of weaned piglets (*p* < 0.05). The fecal microbiota of pregnant sows β diversity reduced and piglet fecal microbiota β diversity increased (*p* < 0.05). Y−dP significantly increased the abundance of Actinobacteria and *Limosilactobacilli* in the feces of pregnant sows (*p* < 0.05), as well as the abundance of Verrucomicrobiota, Bacteroidota, and Fusobacteriota in the feces of piglets (*p* < 0.05). The abundance of Bacteroidota in the feces of pregnant sows is positively correlated with propionic acid (PA) (r > 0.5, *p* < 0.05). The abundance of *Prevotellaceae_NK3B31_group* was positively correlated with Acetic acid (AA), PA, Valerate acid (VA), and total volatile fatty acid (TVFA) in the feces of pregnant sows (r > 0.5, *p* < 0.05), and Bacteroidota and *Prevotellaceae_NK3B31_group* were negatively correlated with the number of stillbirths (r < −0.5, *p* < 0.05). The abundance of *Lactobacillus* and *Holdemanella* in piglet feces was positively correlated with TVFA in feces and negatively correlated with IgA in serum (r > 0.5, *p* < 0.05). In conclusion, supplementing Y−dP to the diet of sows from late gestation to lactation can increase the chao1 index and α diversity of fecal microorganisms in sows during lactation, increase the abundance of Actinobacteria and *Limosilactobacilli* in the feces of sows during pregnancy, and increase the abundance of beneficial bacteria such as Bacteroidetes in piglet feces, thereby improving intestinal health. These findings provide a reference for the application of Y−dP in sow production and a theoretical basis for Y−dP to improve sow production performance.

## 1. Introduction

The intestinal microbiota is a huge community, with trillions of microorganisms living in the intestines of animals. The synergistic relationship between microorganisms and hosts almost exists in various physiological activities, including digestive and metabolic processes and intestinal immunity [1]. Piglets are stressed during weaning due to environmental and dietary changes, which can lead to problems such as reduced feed intake and diarrhea. Recent studies have shown that the main cause of piglet diarrhea is an intestinal flora imbalance [2,3]. Previous studies have found a decrease in the abundance of Lactobacillus in the intestinal microbiome of piglets with diarrhea and a decrease in the number of intestinal microbes [4]. Studies have found that Lactobacillus−rich vaginal and endometrial microbial signatures are associated with better reproductive outcomes [5]. In addition, the microorganisms in the uterus are critical for embryonic development and placenta formation in early pregnancy [6]. This study showed that the abundance of beneficial bacteria in the intestines of sows and offspring piglets increased after feeding fermented bamboo fiber, and there was a certain correlation between beneficial bacteria and the reproductive performance of sows [7]. Therefore, intestinal flora is closely related to sow reproductive performance and piglet diarrhea.

Postbiotics are biologically active products produced by probiotics after processing. Yeast−derived postbiotic (Y−dP) is a microbial product extracted from a specific culture medium by yeast after sufficient anaerobic fermentation. Y−dP contains carbohydrates, amino acids, small peptides, and other nutrients. Supplementing yeast culture in sow feed can improve sow antioxidant capacity and milk production [8]. Other studies have shown that supplementing rabbit diets with postbiotics can improve the quality of rabbit semen [9]. A postbiotic from Aspergillus oryzae can alleviate heat stress by reducing the inflammatory response [10]. Moreover, dietary supplementation with yeast can significantly improve weight gain and feed intake in early−weaned piglets and reduce diarrhea and mortality [11]. Most studies have shown that Y−dP can explain their effects by improving intestinal health [11], immune function [12,13], and nutrient digestibility [14]. However, the effect of supplementing Y−dP with sow diets on gut microbiota in sows and piglets is unclear and needs further study. Therefore, the objectives of this study were to investigate the effects of dietary Y−dP supplementation in late gestation and lactation of sows on the fecal microorganisms of sows and their weaned piglets and the relationship between fecal microorganisms with short−chain fatty acids (SCFA), reproductive performance, and serum cytokines of sows.

## 2. Materials and Methods

The experiment was conducted at the pig farm of the Dekang Agriculture and Animal Husbandry Group Co., Ltd. (Yibin City, China), and this experiment was approved by the Animal Care and Use Committee of Sichuan Agricultural University (ethical approval code: SICAU20211209).

### 2.1. Animals and Experiment Design

A total of 150 Landrace × Yorkshire (parity: 3.93 ± 0.11, backfat thickness: 17.61 ± 0.24 mm) sows at 90 days of gestation were randomly divided into 3 treatment groups (n = 50 per treatment). Sows in the control group (CON) were fed the basal diet (multigrain and multimeal); 0.125% group sows fed the basal diet + Y−dP 1.25 g/kg; 0.2% group sows fed the basal diet + Y−dP 2 g/kg, n = 50. An experimental basal diet was formulated according to the NRC (2012). The composition of the basal diet during pregnancy and lactation is shown in Table S1. During the experiment, no antibiotics or probiotics were used. The supplementary level of Y−dP is based on the manufacturer’s recommendation. The Y−dP (Diamond V, Cedar Rapids, IA, USA) used in this experiment was rich in amino acids, peptides, organic acids, yeast derivative enzymes, and unknown growth factors.

The sows were washed and disinfected at 107 days of gestation and transferred to the farrowing room. Pregnant sows were fed a restricted diet of 3 kg per day from 90 days gestation to 113 days gestation and 3.5 kg per day from 110 days gestation to farrowing. On the day of farrowing, sows were fed 1 kg of feed, followed by an increase of 1 kg of feed per day until the maximum feed intake was reached, and sows were allowed to freely drink during the experiment.

### 2.2. Fecal Sample Collection

In order to compare the differences in fecal microorganisms of sows supplemented with Y−dP during gestation and lactation compared with the CON group. Select 8 sows from each treatment group and collect their feces on days 110 of pregnancy and 21 of lactation, and offspring weaned piglet feces on the day of weaning. During fecal collection, sterile disposable gloves are worn to stimulate the rectum of sows and piglets. The collected feces are placed in a sterilized cryopreservation tube, frozen in liquid nitrogen, and transferred to a −80 °C refrigerator for measurement.

### 2.3. Determination of SCFA Acids in Feces

The concentration of SCFA in the feces was determined using gas chromatography. The steps are roughly as follows: 0.5 g of feces was dissolved in 1.5 mL of distilled water, mixed well, and centrifuged at 3000× *g* for 15 min at 4 °C; 1 mL of supernatant was mixed with 0.2 mL of metaphosphoric acid and 23.3 μL of crotonic acid and placed at 4 °C for 30 min. Centrifuge again at 4 °C (12,000× *g*) for 10 min, and then mix 0.3 mL of supernatant with 0.9 mL of methanol. It was then analyzed by gas chromatography (Varian CP−3800 GC, Palo Alto, CA, USA).

### 2.4. Fecal Microbiological Analysis

The Cetyltrimethylammonium Bromide (CTAB) method was used to extract DNA from feces. Then, use 1% agarose gel electrophoresis to check the purity and concentration of the DNA. Specific primer 515F (CCTAYGGGRBGCASCAG) hypervariable region. All PCR reactions were performed using 15 µL PCR Master Mix (England Biolabs), 2 µM forward and reverse primers, and approximately 10 ng template DNA. PCR products were purified using a gel extraction kit (Qiagen, Germany). Finally, the library was sequenced on the Illumina NovaSeq platform, generating 250 bp paired−end reads.

The Uparse algorithm (Uparse v7.0.1001) were used for all samples of all valid data. The sequences were clustered as operational taxonomic units with 97% consistency. Species annotation of OTU sequences was performed using the Mothur method and the SSUrRNA database of SILVA138.1 (http://www.arb−silva.de/, accessed on 21 June 2023) for species annotation analysis, with the threshold set to 0.8~1.

### 2.5. Statistical Analysis

The data from SCFA were analyzed with MIXED procedures in SAS (V9.4, SAS Institute Inc., Cary, NC, USA). The Spearman method were used to analyze the correlation between microorganisms and relevant data. The reproductive data and cytokine data involved in the correlation analysis in the present study have been published online [13]. All data are shown as mean ± standard error. *p* < 0.05 is considered a significant difference; 0.05 ≤ *p* < 0.10 indicates a significant trend; and correlation coefficients r ≥ 0.5 and r ≤ −0.5 indicate correlation.

## 3. Results

### 3.1. Effect of Y−dP Supplementation in the Sow Diet on Fecal SCFA in the Feces of Sows during Gestation, Lactation, and Piglets

On 110 days of gestation, the concentration of isobutyric acid (IBA) in 0.125% group feces was significantly higher than that in the CON group (*p* < 0.05, Table 1). The butyric acid (BA) content in the feces of weaned piglets in the 0.125% group was significantly lower than that in the CON group (*p* < 0.05, Table 1), and the content of IBA in the 0.125% group tended to decrease compared with the CON group (*p* = 0.08, Table 1). There was no significant difference in the content of other SCFA among treatment groups (*p* > 0.05, Table 1).

### 3.2. Fecal Microbial Species Diversity Analysis of Pregnant Sows, Lactating Sows, and Weaned Piglets

#### 3.2.1. Fecal Microbial Species Diversity Analysis of Pregnant Sows

**Figure 1:**
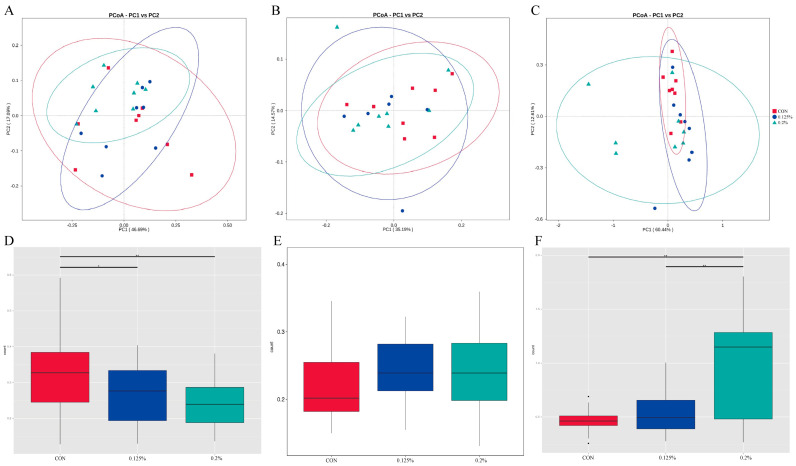
Effects of Y−dP supplementation in sow diet on fecal microbial β diversity during gestation (**A**,**D**), lactation (**B**,**E**) of sows and weaned piglets (**C**,**F**) (**A**–**C**) are the principal coordinate analysis (PCoA) plots of the three groups; (**D**–**F**) are the differences in microbial community β diversity among the three groups; CON: basal diet; 0.125%: basal diet +1.25 g/kg Y−dP; 0.2%: Basal diet +2 g/kg Y−dP. n = 8. **, *p* < 0.01, *, 0.01 < *p* ≤ 0.05.

In α diversity, compared with the CON group, the fecal chao1 index of pregnant sows in the 0.2% group tended to increase (*p* = 0.08), while there was no significant difference in other diversity indexes (*p* > 0.05, Table 2). The β diversity analysis showed that the microbial community composition of the CON group was significantly different from that of the 0.125% and 0.2% groups (Figure 1A,D).

#### 3.2.2. Fecal Microbial Species Diversity Analysis of Lactating Sows

In α diversity, the chao 1 index of the 0.2% group was higher than that of the 0.125% group (*p* < 0.05, Table 2), but there was no significant difference in other diversity indexes (*p* > 0.05, Table 2). There was no significant difference in microbial β diversity among the three groups (*p* > 0.05, Figure 1B,E).

#### 3.2.3. Fecal Microbial Species Diversity Analysis of Weaned Piglets

In α diversity, the observed species in the feces of piglets in the CON group significantly increased compared to the 0.2% group (*p* < 0.05, Table 2); and the chao1 index in the CON group showed an increasing trend compared with the 0.2% group (*p* < 0.05, Table 2), while other diversity index showed no significant difference (*p* > 0.05, Table 2). β diversity results showed that the microbial community composition of the 0.2% group was significantly different from that of the CON group and the 0.125% group (*p* < 0.05, Figure 1C,F).

### 3.3. Relative Abundance of Fecal Microorganisms in Pregnant Sows, Lactating Sows, and Weaned Piglets

#### 3.3.1. Relative Abundance of Fecal Microorganisms in Pregnant Sows

**Figure 2:**
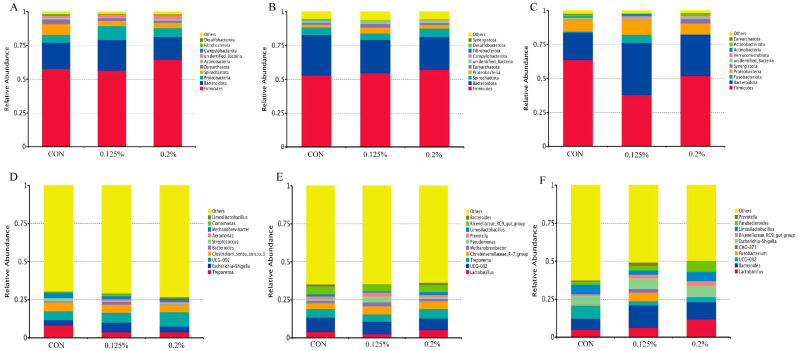
Effects of Y−dP supplementation in the sow diet on the relative fecal microbial abundance of sows during gestation (**A**,**D**), lactation (**B**,**E**), and weaned piglets (**C**,**F**). (**A**–**C**) are the relative abundances at phylum level; (**D**–**F**) are the relative abundances at genus level; CON: basal diet; 0.125%: basal diet +1.25 g/kg Y−dP; 0.2%: Basal diet +2 g/kg Y−dP. n = 8.

Firmicutes and Bacteroidota dominate the phylum level. The abundance of Actinobacteria in groups 0.125% and 0.2% was significantly higher than that in the CON group (*p* < 0.05, Figure 2A and Table S2). Compared with the CON group, the abundance tended to decrease (*p* = 0.08, Figure 2A and Table S2). At the genus level, UCG−002 in the 0.2% group was significantly higher than that in the CON group and the 0.125% group (*p* < 0.05, Figure 2D and Table S3). Bacteroides in the CON group and the 0.125% group were significantly higher than those in the 0.2% group (*p* < 0.01; Figure 2D and Table S3). In the CON group, the abundance of Methanobrevibacter increased (*p* = 0.08, Figure 2D and Table S3). The abundance of Limosilactobacillus in groups 0.125% and 0.2% was significantly higher than that in the CON group (*p* < 0.05, Figure 2D and Table S3).

#### 3.3.2. Relative Abundance of Fecal Microorganisms in Lactating Sows

Firmicutes and Bacteroidetes dominate fecal microorganisms in lactating sows. Compared with the 0.125% group, the abundance of Euryarchaeota in the CON group and 0.2% group tended to increase (*p* = 0.07, Figure 2B and Table S2), and the abundance of Synergistota in the 0.125% group tended to decrease (*p* = 0.06, Figure 2B and Table S2) compared with the CON group and 0.2% group. At the genus level, compared with the 0.125% group, the abundance of Methanobrevibacter in the 0.2% group and CON group had an increasing trend (*p* = 0.05, Figure 2E and Table S3). The abundance of Prevotella in the 0.2% group was significantly lower than that in the CON group (*p* < 0.05, Figure 2E and Table S3). The abundance of Lactobacillus and Limosilactobacillus in the CON group tended to increase compared with the other two groups (*p* = 0.07, *p* = 0.06, Figure 2E and Table S3).

#### 3.3.3. Relative Abundance of Fecal Microorganisms in Weaned Piglets

Firmicutes and Bacteroidetes dominate the fecal microorganisms of weaned piglets. The abundance of Firmicutes in the CON group was significantly increased compared with that in the 0.125% group (*p* < 0.01, Figure 2C and Table S2). The abundance of Fusobacteriota in the 0.125% group was significantly higher than that in the CON group and the 0.2% group (*p* < 0.05, Figure 2C and Table S2). Compared with the CON group and 0.2% group, the abundance of Actinobacteriota in the 0.125% group showed a trend of decreasing (*p* = 0.09, Figure 2C and Table S2). Compared with the CON group, the abundance of Bacteroidota and Verrucomicrobacteria in the 0.125% group was significantly increased (*p* < 0.05, Figure 2C and Table S2). The abundance of Synergistota in the 0.2% group tended to increase compared with the CON group and the 0.125% group (*p* = 0.06, Figure 2C and Table S2), and the abundance of Euryarchaeota was significantly increased compared with that in the 0.125% group (*p* < 0.05, Figure 2C and Table S2). At the genus level, compared with the CON group, the abundance of Bacteroides in the 0.125% and 0.2% groups tended to increase (*p* = 0.08, Figure 2F and Table S3), but decreased the abundance of UCG−002 (*p* = 0.07, Figure 2F and Table S3). The abundance of Fusobacterium in the 0.125% group was significantly higher than that in the CON group and the 0.2% group (*p* < 0.05, Figure 2F and Table S3). The abundance of Rikenellaceae_RC9_gut_group in the 0.2% group was higher than that in the CON group (*p* = 0.09, Figure 2F and Table S3). Compared with the CON group and the 0.125% group, the abundance of Parabacteroides in the 0.2% group was significantly increased (*p* < 0.05, Figure 2F and Table S3), and the abundance of Prevotella had a tendency to decrease (*p* = 0.05, Figure 2F and Table S3).

### 3.4. Correlation Analysis

#### 3.4.1. Correlation Analysis of Fecal Microorganisms: Phylum Level and Genus Level with Fecal SCFA in Pregnant Sows

**Figure 3:**
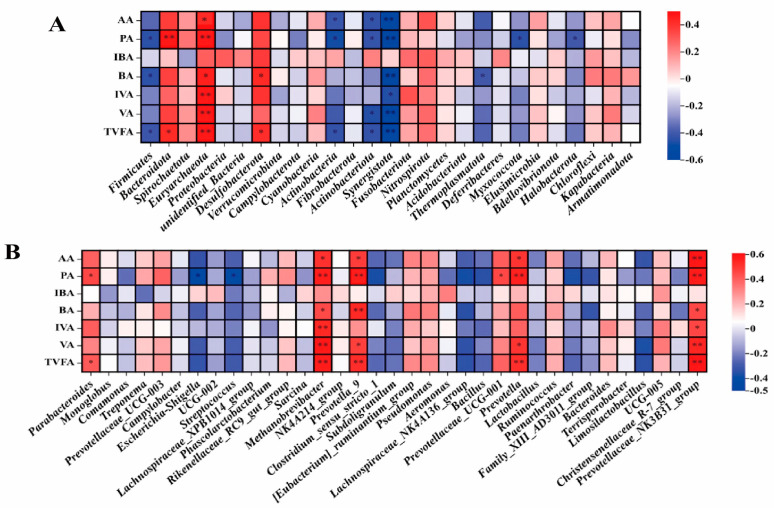
Correlation analysis between SCFA in feces and phylum (**A**) and genus (**B**) levels of fecal microorganisms in pregnant sows. Acetic acid: AA; Propionic acid: PA; Isobutyric acid: IBA; Butyric acid: BA; Isovalerate acid: IVA; Valerate acid: VA; Total volatile fatty acids: TVFA. n = 8. The value corresponding to the color of the middle heat map is the correlation coefficient r, which ranges from −1 to 1. r < 0 is a negative correlation; r > 0 is a positive correlation. **, *p* < 0.01, *, 0.01 < *p* ≤ 0.05.

At the phylum level of fecal microorganisms in pregnant sows (Figure 3A), Propionic acid (PA) was positively correlated with the abundance of Bacteroidota (r > 0.5, *p* < 0.01). Acetic acid (AA), PA, Isovalerate acid (IVA), Valerate acid (VA), and Total volatile fatty acid (TVFA) were positively correlated with the abundance of Euryarchaeota (r > 0.5, *p* < 0.01). AA, VA, and TVFA were negatively correlated with the abundance of Synergistota (r < −0.5, *p* < 0.01). At the genus level (Figure 3B), AA, PA, IVA, VA, and TVFA were positively correlated with the abundance of *Methanobrevibacter* (r > 0.5, *p* < 0.01). PA, BA, and TVFA were positively correlated with the abundance of *Prevotella_9* (r > 0.5, *p* < 0.01). AA, PA, VA, and TVFA were positively correlated with the abundance of *Prevotellaceae_NK3B31_group* (r > 0.5, *p* < 0.01).

#### 3.4.2. Correlation Analysis of Fecal Microorganisms: Phylum Level and Genus Level with Fecal SCFA in Weaned Piglets

**Figure 4:**
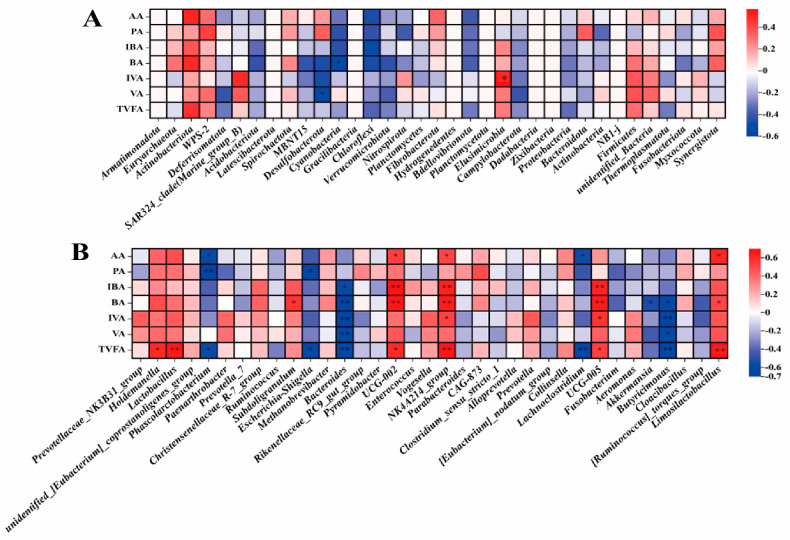
Correlation analysis between SCFA in feces and phylum (**A**) and genus (**B**) levels of fecal microorganisms in weaned piglets. Acetic acid: AA; Propionic acid: PA; Isobutyric acid: IBA; Butyric acid: BA; Isovalerate acid: IVA; Valerate acid: VA; Total volatile fatty acid TVFA. n = 8. The value corresponding to the color of the middle heat map is the correlation coefficient r, which ranges from −1 to 1. r < 0 is a negative correlation; r > 0 is a positive correlation. **, *p* < 0.01, *, 0.01 < *p* ≤ 0.05.

At the phylum level (Figure 4A), BA is negatively correlated with the abundance of Cyanobacteria (r = −0.5, *p* < 0.05), VA is negatively correlated with the abundance of Desulfobacterota (r > 0.5, *p* < 0.05), and IVA is positively correlated with the abundance of Elusimicrobia (r > 0.5, *p* < 0.05). At the genus level (Figure 4B), TVFA was positively correlated with the abundance of *Holdemanella* (r > 0.5, *p* < 0.05). There was a positive correlation between TVFA and the abundance of *Lactobacillus* (r > 0.5, *p* < 0.01). AA, PA, and TVFA were negatively correlated with the abundance of *Phascolarctobacterium* (r < −0.5, *p* < 0.05). PA and TVFA were negatively correlated with the abundance of *Escherichia−Shigella* (r < −0.5, *p* < 0.05), and IBA, BA, IVA, VA, and TVFA were negatively correlated with the abundance of *Bacteroides* (r < −0.5, *p* < 0.05). AA, IBA, BA, and TVFA were positively correlated with the abundance of *UCG−002* (r > 0.5, *p* < 0.05), AA, IBA, BA, IVA, and TVFA were positively correlated with the abundance of *NK4A214_group* (r > 0.5, *p* < 0.05), and AA and TVFA are negatively correlated with the abundance of *Lachnoclostridium* (r < −0.5, *p* < 0.05). IBA, BA, IVA, and TVFA were positively correlated with the abundance of *UCG−005* (r > 0.5, *p* < 0.05). IBA, BA, IVA, and TVFA were negatively correlated with the abundance of *Butyricimonas* (r < −0.5, *p* < 0.05), while AA, BA, and TVFA were positively correlated with *Limosilactobacillus* (r > 0.5, *p* < 0.05).

#### 3.4.3. Correlation Analysis of Fecal Microorganisms: Phylum Level and Genus Level with Serum Cytokines in Pregnant Sows

**Figure 5:**
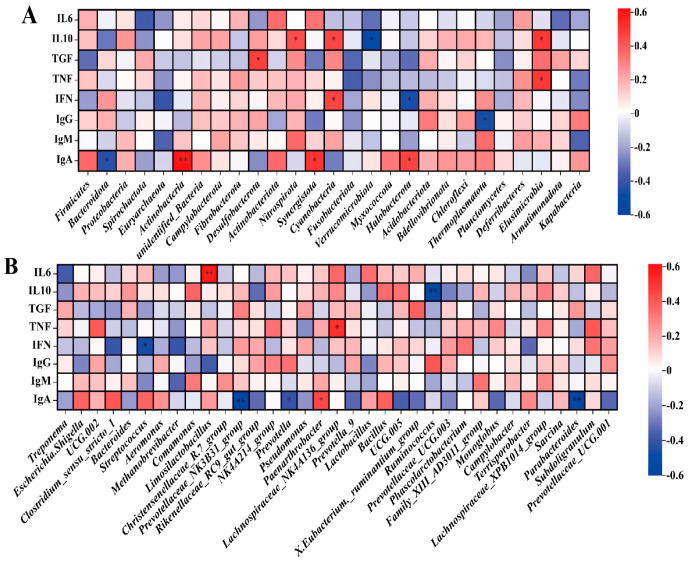
Correlation analysis between serum cytokines and phylum (**A**) and genus (**B**) levels of fecal microorganisms in pregnant sows. IgG, Immunoglobulin G; IgM, Immunoglobulin M; IgA, Immunoglobulin A; IL10, Interleukin 10; IL6, Interleukin 6; TNF, tumor necrosis factor α; IFN, interferon γ; TGF, transforming growth factor β. n = 8. The value corresponding to the color of the middle heat map is the correlation coefficient r, which ranges from −1 to 1. r < 0 is a negative correlation; r > 0 is a positive correlation. **, *p* < 0.01, *, 0.01 < *p* ≤ 0.05.

The phylum level of pregnant sows’ fecal microorganisms (Figure 5A), the abundance of Actinobacteria, and Synergistota were positively correlated with serum cytokine IgA (r > 0.5, *p* < 0.01). At the genus level (Figure 5B), the abundance of *Limosilactobacillus* was positively correlated with serum cytokine interleukin−6 (IL−6) (r > 0.5, *p* < 0.01), and the abundance of *Lachnospiraceae_NK4A136_group* was positively correlated with cytokine tumor necrosis factor α (TNF−α) (r > 0.5, *p* < 0.01). The abundance of *Prevotellaceae_NK3B31_group* was negatively correlated with serum cytokine immunoglobulin A (IgA) (r < −0.5, *p* < 0.01), and the abundance of *Ruminococcus* was negatively correlated with serum cytokine interleukin−10 (IL−10) (r < −0.5, *p* < 0.01).

#### 3.4.4. Correlation Analysis of Fecal Microorganisms: Phylum Level and Genus Level with Serum Cytokines in Weaned Piglets

**Figure 6:**
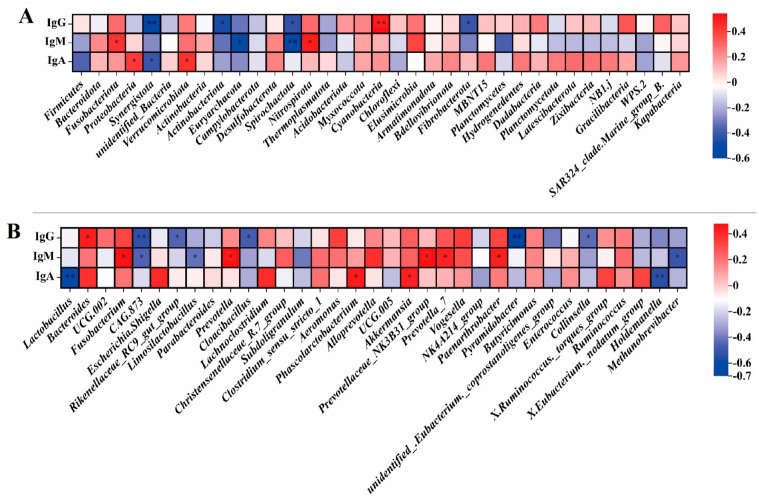
Correlation analysis between serum cytokines and phylum (**A**) and genus (**B**) levels of fecal microorganisms in weaned piglets. IgG, Immunoglobulin G; IgM, Immunoglobulin M; IgA, immunoglobulin A; n = 8. The value corresponding to the color of the middle heat map is the correlation coefficient r, which ranges from −1 to 1. r < 0 is a negative correlation; r > 0 is a positive correlation. **, *p* < 0.01, *, 0.01 < *p* ≤0.05.

At the phylum level (Figure 6A), the abundance of Synergistota was negatively correlated with serum cytokine immunoglobulin G (IgG) (r < −0.5, *p* < 0.01), while the abundance of Cyanobacteria was positively correlated with serum cytokine IgG (r > 0.5, *p* < 0.01). The abundance of Euryarchaeota and Spirochaetota was negatively correlated with the serum cytokine IgM (r < −0.5, *p* < 0.05). At the genus level (Figure 6B), there was a negative correlation between the abundance of *CAG 873* and IgG (r < −0.5, *p* < 0.01). The abundance of *Methanobrevibacter* was negatively correlated with serum cytokine IgM (r = −0.50, *p* < 0.05), and the abundance of *Lactobacillus* and *Holdemanella* was negatively correlated with serum cytokine IgA (r < −0.5, *p* < 0.01).

#### 3.4.5. Correlation Analysis of Fecal Microorganisms and Reproductive Performance of Pregnant Sows

**Figure 7:**
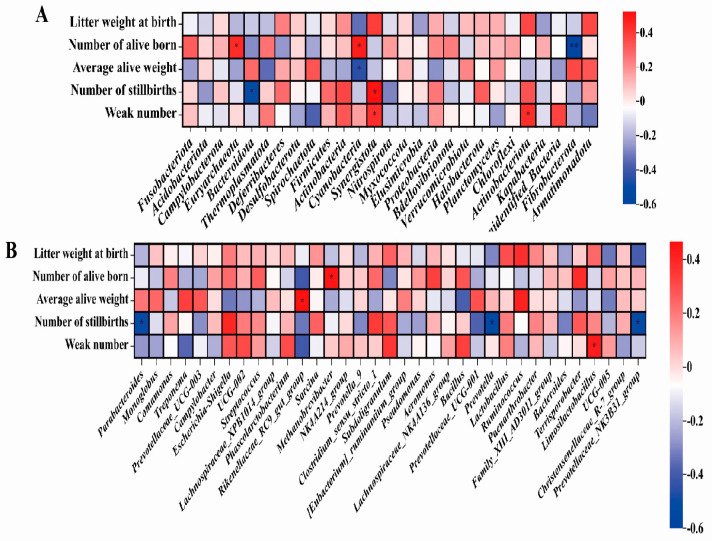
Correlation analysis of the farrowing performance of pregnant sows with fecal microorganisms at phylum level (**A**) and genus level (**B**), n = 8. The value corresponding to the color of the middle heat map is the correlation coefficient r, which ranges from −1 to 1. r < 0 is a negative correlation; r > 0 is a positive correlation. **, *p* < 0.01, *, 0.01 < *p* ≤ 0.05.

In the analysis of the correlation between fecal microorganisms and the reproductive performance of pregnant sows, at the phylum level (Figure 7A), the number of live births was negatively correlated with the abundance of Fibrobacterota (r < −0.5, *p* < 0.01). The number of stillbirths was negatively correlated with the abundance of Bacteroidota and positively correlated with the abundance of Synergistota (r = −0.5, 0.5, *p* < 0.05). The number of stillbirths was negatively correlated with the abundance of *Parabacteroides*, *Prevotella*, and *Prevotellaceae_NK3B31_group* at the genus level (r = −0.56, −0.5, −0.51, *p* < 0.05, Figure 7B).

## 4. Discussion

SCFA in the intestine is mainly produced by the fermentation of carbohydrates that are not easily digested by intestinal microorganisms. SCFA is not only an important energy source for intestinal epithelial cells but also has a protective effect on intestinal barrier function and prevents intestinal inflammation [15,16]. In this study, our detection of SCFA in sow feces showed that the content of IBA in the feces of the 0.125% group on the 110th day of pregnancy was significantly higher than that of the CON group. IBA is generated from albumin and valine and reflects the level of protein degradation in the colon [17]. Similar to this study, supplementation with live yeast and mannan increased the level of IBA in bovine rumen [18]. It suggests that Y−dP may affect the utilization of amino acids by sows. Correlation analysis showed that *Methanobrevibacter* in the feces of pregnant sows was positively correlated with AA, PA, IVA, VA, and TVFA, and the abundance of *Methanobrevibacter* showed a decreasing trend after feeding Y−dP. *Methanobrevibacter* is a type of methanogenic bacteria that uses SCFA to produce methane by decomposing nutrients in feed, causing feed energy loss and environmental pollution [19]. The significant reduction of Methanogens in sow feces after adding Y−dP indicates that Y−dP can reduce methane production and thereby reduce energy loss. The content of BA and IBA in the feces of 0.125% piglets also decreased compared to the CON group. SCFA is an important energy source for colon epithelial cells, and butyrate can increase the expression of Claudin−1 in the intestine and improve the intestinal barrier, which is extremely important for maintaining intestinal health [20,21]. The decrease in BA and IBA in feces indicates that the 0.125% group absorbed more BA and IBA than the CON group. Our previous research results showed that Y−dP can reduce diarrhea in piglets, and Espinosa et al. have shown that yeast can improve intestinal nutrient utilization efficiency [13,22]. Therefore, we speculate that yeast culture can improve intestinal health and reduce diarrhea rates by improving the absorption of SCFA in the hindgut.

The animal gut contains huge microbial flora, which can regulate the host’s digestion and metabolism [23,24], immunity [25,26], and other physiological activities. The microorganisms in feces can, to some extent, reflect the status of the host’s intestinal microbiota [27]. Previous studies have shown that intestinal diseases are often associated with a lower abundance of gut microbiota [28,29]. In this study, the 0.2% group increased the chao1 index of fecal microbial and α diversity of lactating sows compared with the CON group and 0.125% group and had a tendency to increase the chao1 index of pregnant sows. Supplementing yeast hydrolysates to the diet of sows during pregnancy can improve the gut microbiota of sows, thereby improving the sow’s physiological condition and the intestinal health of offspring [30]. Therefore, supplementing Y−dP with the diet can improve the intestinal health of sows by increasing the abundance of gut microbiota. However, in piglets, we found that the fecal observed species index of the 0.2% group was significantly lower than that of the CON group, and the chao1 index tended to decrease, which might be due to the fact that the intestinal microorganisms of piglets were susceptible to external influences in the early stages, and too high Y−dP supplementation in sows would affect the colonization of the microflora of piglets. Our previous study has shown that supplementing Y−dP with sow feed can increase weaning weight and reduce mortality and diarrhea rates in piglets [13]. This may be related to the improvement of gut microbiota abundance by Y−dP. However, β diversity analysis showed that supplementing Y−dP to the diet reduced fecal microbiota in pregnant sows β diversity, significantly improving weaned piglet fecal microbiota β diversity. As pregnancy progresses, the diversity of intestinal microbes in sows will decrease [31,32], and the decrease of microbial diversity in late pregnancy has a positive effect on maternal metabolism and the maintenance of pregnancy [32]. However, the increase in microbial diversity in the intestinal tract of weaned piglets is beneficial to enhance intestinal barrier function, maintain intestinal health, and reduce diarrhea rates [33,34]. Therefore, the supplementation of Y−dP in sow diets can improve intestinal health by regulating the diversity of intestinal flora in sows and weaned piglets.

The results of this study show that the fecal microorganisms of sows during pregnancy, lactation, and offspring piglets are mainly Firmicutes and Bacteroidota, which is consistent with previous results [31]. Supplementing Y−dP to the diet did not alter the dominant phylum of gut microbiota in sows and weaned piglets. The supplementation of Y−dP increased the abundance of Actinobacteria in pregnant sows, with a trend towards reducing the abundance of Euryarchaeota, but it increased the abundance of Euryarchaeota in lactating sows, and we found a positive correlation between Actinobacteria and serum IgA in pregnant sows. Studies have found that Actinobacteria are associated with the improvement of the intestinal barrier and intestinal immunity [35], while Euryarchaeota are associated with sow obesity [36]. During pregnancy, excessive obesity in sows can have an adverse effect on delivery, while during lactation, the high synthesis of breast milk can significantly reduce body weight and backfat. The supplementation of Y−dP with diet may reduce weight loss in sows by regulating the gut microbiota, which is consistent with our previous research results [13]. Our research results indicate that the supplementation of Y−dP has a trend of reducing the abundance of Methanobrevibacter in the feces of pregnant sows and significantly increasing the abundance of *Limosilactobacillus*, and *Limosilactobacillus* is positively correlated with IL−6 in serum. *Limosilactobacillus* has been shown to have antibacterial and intestinal immunity−enhancing effects and can reduce inflammatory responses and prevent the occurrence of colitis by regulating the NF−κB signaling pathway [37]. Therefore, Y−dP may inhibit intestinal inflammation by enhancing *Limosilactobacillus* and then enhancing intestinal immunity. However, the supplementation of Y−dP to the diet had a tendency to increase the abundance of *Methanobrevibacter* and reduce the abundance of *Limosilactobacillus* in the feces of lactating sows. *Metanobrevibacter* is related to energy absorption in the intestine [38]. This may be due to the stable physiological activity of sows after delivery, which improves energy utilization and reduces the occurrence of inflammation. Our study results in piglet feces showed that the 0.125% group significantly reduced Firmicutes compared to the control group and increased the abundance of Bacteroidota, Fusobateriota, and Verrucomicrobiota in the feces. As we all know, Bacteroidota and Verrucomicrobiota are beneficial bacteria that contribute to intestinal metabolism and the maintenance of homeostasis [39,40,41]. Therefore, we conclude that although Y−dP reduces the abundance of Firmicutes, it increases the abundance of other beneficial bacteria to maintain intestinal health. At the genus level, the supplementation of Y−dP to the sow diet significantly increased the abundance of beneficial bacterial genera in piglet feces, including *Bacteroides*, *Rikenellaceae_RC9_gut_group*, and *Parabacteroides*. These beneficial bacteria are essential for the intestinal homeostasis and immunity of piglets [42,43]. Fusobacterium is mainly associated with intestinal inflammation and colon cancer and is a pathogenic bacterium. In this study, the 0.125% group increased the abundance of the Fusobacterium compared to the CON group and 0.2% group, and we also did not find any intestinal diseases in the 0.125% group of weaned piglets. This may be related to the amount of Y−dP added; 0.125% promoted the growth of the Fusobacterium, while 0.2% inhibited the growth of the Fusobacterium. Moreover, our previous research has shown that Y−dP has the effect of reducing diarrhea rates in weaned piglets [13], which may be related to the improvement of gut microbiota in weaned piglets by Y−dP, similar to previous studies [44,45].

Studies have shown that gut microbes affect every stage and level of human reproduction, including the maturation of follicles and oocytes in the ovaries, fertilization, and embryo migration, implantation, throughout pregnancy, and even during delivery [46], and some of the microbes in the gut are found in the uterus and placenta [6,47]. The correlation analysis results showed that the number of live births is negatively correlated with the abundance of Fibrobacteria. Fibrobacteria is related to the digestion and absorption of fiber, and studies have shown that dietary fiber can have an effect on the reproductive performance of sows [48,49]. It suggests that the reproductive performance of sows may be related to fiber metabolism. In addition, the number of stillbirths was negatively correlated with *Parabacteroides*, *Prevotella*, and *Prevotellaceae_NK3B31_group*. *Prevotella* is crucial for lipid, carbohydrate, and protein metabolism [50,51], while it can improve intestinal immunity and have anti−inflammatory properties [52]. Indicating that the health of the intestines and the metabolism of substances in the intestines can also affect fetal development.

## 5. Conclusions

In conclusion, supplementation of Y−dP in the late gestation and lactation diets of sows can increase the fecal microbial chao1 index and α diversity of lactating sows, increase beneficial microorganisms in the feces of pregnant sows and weaned piglets, thus helping to improve the homeostasis of the intestinal microbiota and regulate the generation of SCFA, which plays a crucial role in promoting intestinal health. In addition, improved reproductive performance in sows is related to the improvement of fecal flora and immune function.

## Figures and Tables

**Table 1:** Effect of Y−dP supplementation in the sow diet on fecal SCFA acids in the feces of sows during gestation and lactation and weaned piglets.

Items	CON	0.125%	0.2%	*p*-Values
Day 113 of gestation				
AA	3.08 ± 0.42	2.79 ± 0.14	2.38 ± 0.14	0.24
PA	0.88 ± 0.16	0.89 ± 0.07	0.67 ± 0.04	0.31
IBA	0.13 ± 0.01 ^b^	0.19 ± 0.02 ^a^	0.17 ± 0.01 ^ab^	0.03
BA	0.55 ± 0.12	0.62 ± 0.07	0.39 ± 0.07	0.20
IVA	0.27 ± 0.01	0.30 ± 0.03	0.25 ± 0.02	0.22
VA	0.15 ± 0.01	0.14 ± 0.02	0.11 ± 0.01	0.11
TVFA	5.03 ± 0.73	5.14 ± 0.41	4.28 ± 0.43	0.48
Day 21 of lactation				
AA	2.77 ± 0.12	2.74 ± 0.18	2.99 ± 0.32	0.88
PA	0.92 ± 0.04	0.99 ± 0.09	1.08 ± 0.11	0.58
IBA	0.22 ± 0.02	0.21 ± 0.02	0.18 ± 0.01	0.47
BA	0.39 ± 0.02	0.43 ± 0.04	0.47 ± 0.7	0.52
IVA	0.26 ± 0.01	0.29 ± 0.03	0.27 ± 0.02	0.66
VA	0.13 ± 0.01	0.14 ± 0.01	0.14 ± 0.01	0.97
TVFA	4.69 ± 0.19	4.80 ± 0.34	5.16 ± 0.50	0.63
Weaned piglets				
AA	1.39 ± 0.25	0.83 ± 0.20	1.13 ± 0.26	0.32
PA	0.33 ± 0.05	0.26 ± 0.09	0.39 ± 0.09	0.54
IBA	0.16 ± 0.03	0.06 ± 0.02	0.10 ± 0.03	0.08
BA	0.49 ± 0.18 ^a^	0.10 ± 0.03 ^b^	0.22 ± 0.06 ^ab^	0.04
IVA	0.35 ± 0.06	0.21 ± 0.11	0.16 ± 0.03	0.11
VA	0.10 ± 0.03	0.11 ± 0.08	0.07 ± 0.02	0.37
TVFA	2.81 ± 0.37	1.57 ± 0.32	2.07 ± 0.43	0.12

Data are expressed as mean ± standard error. CON group: basal diet; 0.125% group: basal diet +1.25 g/kg Y−dP; 0.2% group: basal diet +2 g/kg Y−dP. Acetic acid: AA; Propionic acid: PA; Isobutyric acid: IBA; Butyric acid: BA; Isovalerate acid: IVA; Valerate acid: VA; Total volatile fatty acid TVFA. ^a, b^ Different superscript lowercase letters indicate significant differences (*p* < 0.05). n = 8.

**Table 2:** The effects of Y−dP supplementation in the sow diet affect the fecal microbial α diversity index of pregnant sows, lactating sows, and weaned piglets.

Items	CON	0.125%	0.2%	*p*-Values
Pregnant sows				
Observed species	916.00 ± 14.64	924.13 ± 19.67	954.86 ± 13.61	0.25
Shannon	7.03 ± 0.11	7.21 ± 0.09	7.15 ± 0.07	0.39
Simpson	0.98 ± 0.00	0.98 ± 0.00	0.98 ± 0.00	0.53
Chao 1	970.56 ± 17.24	974.44 ± 19.32	1032.35 ± 23.76	0.08
ACE	976.22 ± 16.45	978.45 ± 18.94	1036.30 ± 22.87	0.13
Lactating sows				
Observed species	973.38 ± 26.25	939.50 ± 23.86	1007.38 ± 21.28	0.18
Shannon	7.17 ± 0.08	7.04 ± 0.06	7.25 ± 0.06	0.14
Simpson	0.98 ± 0.00	0.98 ± 0.00	0.98 ± 0.00	0.20
Chao 1	1050.12 ± 17.77 ^ab^	999.63 ± 26.93 ^b^	1083.77 ± 21.47 ^a^	0.048
ACE	1038.11 ± 28.02	1005.23 ± 29.27	1088.01 ± 23.53	0.13
Piglets				
Observed species	844.00 ± 57.05 ^a^	730.88 ± 43.04 ^ab^	653.38 ± 20.33 ^b^	0.02
Shannon	6.15 ± 0.27	5.88 ± 0.23	5.45 ± 0.12	0.17
Simpson	0.96 ± 0.01	0.94 ± 0.01	0.94 ± 0.01	0.30
Chao 1	909.34 ± 63.96 ^a^	805.65 ± 52.64 ^ab^	721.99 ± 22.70 ^b^	0.09
ACE	920.85 ± 68.29	812.64 ± 52.42	735.04 ± 25.11	0.12

Data are expressed as mean ± standard error. CON group: basal diet; 0.125% group: basal diet +1.25 g/kg Y−dP; 0.2% group: basal diet +2 g/kg Y−dP. ^a, b^ Different superscript lowercase letters indicate significant differences (*p* < 0.05). n = 8.

## Data Availability

The data presented in this study are available in this article.

## Supplementary Materials

The following supporting information can be downloaded at: https://www.mdpi.com/article/10.3390/ani14010162/s1. Table S1: Composition and nutrient level of the experimental basal diet; Table S2: Effects of Y−dP supplementation in sow diet on fecal microbial phylum level distribution in pregnant sows, lactating sows, and weaned piglets; Table S3: Effects of Y−dP supplementation in sow diet on fecal microbial genus level distribution in pregnant sows, lactating sows, and weaned piglets.
