# Supplementary material for: Microbial Mechanistic Insight into the Role of Yeast−Derived Postbiotics in Improving Sow Reproductive Performance in Late Gestation and Lactation Sows
Source: Animals (Basel). 2024 Jan 4;14(1):162. doi: 10.3390/ani14010162 (PMC10777949; doi:10.3390/ani14010162)
Supplement: Supplementary file 1 [file animals-14-00162-s001.zip › Table S1.pdf]

Table S1 Composition and nutrient level of experimental basal diet

Items	Pregnancy	Lactation
Ingredient, %		
Corn	20.00	29.96
Wheat	13.72	--
Wheatmeal, 14%CP	15.00	15.00
Soybean meal, 46%CP	6.90	8.70
Fermented soybean meal	--	5.80
Expanded soybean	--	5.00
Brown rice mix	10.00	10.00
Sugar beet meal granulate	6.00	--
Rice bran meal	10.00	14.90
Rice bran	5.00	5.00
Soybean skin	7.80	--
Soybean oil	1.55	2.00
Calcium hydrogen phosphate type I	1.75	1.31
Limestone	0.91	1.15
Sodium chloride	0.35	0.35
L-Lys HCl, 98%	0.12	0.12
DL-Met, 98.5%	0.05	0.05
L-Thr, 98%	0.02	0.02
L-Trp, 98%	0.02	0.02
Sodium bicarbonate	0.09	--
Choline chloride, 50%	0.08	0.08
Vitamin and mineral premix[1]	0.64	0.54
Total	100.00	100.00
Nutrient level[2]		
Crude protein, %	13.50	17.00
Calcium, %	0.90	0.85
Total phosphorus, %	0.70	0.60

[1]Per kilogram of diet provided: Cu 20 mg; I 0.4 mg; Fe 110 mg; Mn 40 mg; Se 0.25 mg; Zn 115 mg; VA 10000 IU; VB_1 6.0 mg; VB_2 5.0 mg; VB_6 4.0 mg; VB_{12} 30 μg; VD_3 2200 IU; VE 80 IU; VK_3 4.0 mg; Biotin 0.3 mg; Folic acid 3.0 mg; Pantothenic acid 20 mg; Nicotinic acid 40 mg.

[2] Crude protein is calculated values, total phosphorus and calcium are measured values.
