# Supplementary material for: Microbial Mechanistic Insight into the Role of Yeast−Derived Postbiotics in Improving Sow Reproductive Performance in Late Gestation and Lactation Sows
Source: Animals (Basel). 2024 Jan 4;14(1):162. doi: 10.3390/ani14010162 (PMC10777949; doi:10.3390/ani14010162)
Supplement: Supplementary file 1 [file animals-14-00162-s001.zip › Table S2.pdf]

Table S2. Effects of Y-dP supplementation in sow diet on fecal microbial phylum level distribution in pregnant sows, lactating sows and weaned piglets. (%)

Items	CON	0.125%	0.2%	*P*-values
Pregnant sows				
Firmicutes	57.86±4.58	56.60±1.05	64.93±2.04	0.12
Bacteroidota	19.28±3.52	22.70±2.86	16.45±2.80	0.37
*P*roteobacteria	3.74±1.62	10.25±3.63	6.57±2.32	0.11
Spirochaetota	5.84±1.56	3.83±0.85	3.90±0.89	0.38
Euryarchaeota	3.49±0.88	1.55±0.38	1.73±0.45	0.08
Actinobacteria	0.05±0.00[b]	0.09±0.01[a]	0.08±0.00[a]	0.03
unidentified_Bacteria	2.00±0.18[b]	2.27±0.09[ab]	2.82±0.23[a]	0.01
Campylobacterota	0.20±0.11	0.12±0.03	0.11±0.04	0.90
Fibrobacterota	0.05±0.02	0.03±0.01	0.06±0.01	0.20
Desulfobacterota	0.40±0.04	0.46±0.05	0.35±0.03	0.22
Others	1.68±0.22	1.41±0.11	1.53±0.15	0.51
Lactating sows				
Firmicutes	53.01±1.99	54.75±2.48	57.14±2.71	0.46
Bacteroidota	29.72±2.62	24.36±2.25	24.23±2.02	0.18
Spirochaetota	4.90±0.38	4.86±1.36	6.37±1.43	0.58
*P*roteobacteria	2.01±0.39	4.39±1.94	2.48±0.65	0.82
Euryarchaeota	1.42±0.25	0.98±0.27	1.80±0.09	0.07
unidentified_Bacteria	1.49±0.12	1.81±0.22	1.76±0.12	0.29
Campylobacterota	0.06±0.02	0.03±0.01	0.49±0.26	0.15
Fibrobacterota	0.20±0.08	0.15±0.04	0.08±0.01	0.16
Desulfobacterota	0.44±0.03	0.45±0.08	0.39±0.04	0.75
Synergistota	0.15±0.06	0.01±0.00	0.14±0.06	0.06
Others	5.26±1.28	6.35±1.46	3.74±0.49	0.20
Piglets				
Firmicutes	63.77±4.28[a]	38.10±4.15[b]	51.95±6.12[ab]	<0.01
Bacteroidota	20.05±3.66[b]	38.03±5.44[a]	30.46±4.47[ab]	0.04
Fusobacteriota	0.80±0.26[b]	2.86±1.17[a]	0.13±0.05[b]	0.02
*P*roteobacteria	8.45±1.88	10.70±2.05	8.01±2.34	0.69
Synergistota	0.45±0.15	0.39±0.13	3.58±1.67	0.06
unidentified_Bacteria	1.29±0.15[a]	0.73±0.06[b]	0.74±0.12[b]	0.02
Verrucomicrobiota	0.16±0.05[b]	1.74±0.77[a]	0.40±0.34[ab]	0.01
Actinobacteria	0.25±0.06	1.60±0.75	0.09±0.02	0.33
Actinobacteriota	1.31±0.50	0.25±0.08	0.59±0.15	0.09
Euryarchaeota	0.28±0.13[ab]	0.06±0.03[b]	0.43±0.12[a]	0.02
Others	2.29±0.26	2.01±0.18	1.79±0.31	0.41

Data are expressed as mean ± standard error. CON group: basal diet; 0.125% group: basal diet + 1.25g /kg Y-dP; 0.2% group: basal diet +2 g/kg Y-dP. [a, b] Different superscript lowercase letters indicate significant differences ($P < 0.05$). n=8.
