# Supplementary material for: Microbial Mechanistic Insight into the Role of Yeast−Derived Postbiotics in Improving Sow Reproductive Performance in Late Gestation and Lactation Sows
Source: Animals (Basel). 2024 Jan 4;14(1):162. doi: 10.3390/ani14010162 (PMC10777949; doi:10.3390/ani14010162)
Supplement: Supplementary file 1 [file animals-14-00162-s001.zip › Table S3.pdf]

Table S3. Effects of Y-dP supplementation in sow diet on fecal microbial genus level distribution in pregnant sows, lactating sows and weaned piglets. (%)

Items	CON	0.125%	0.2%	*P*-values
Pregnant sows				
Treponema	5.84±1.56	3.83±0.85	3.90±0.89	0.38
Escherichia-Shigella	3.52±1.46	6.12±2.19	3.51±0.86	0.40
UCG-002	5.92±0.98[b]	6.59±1.12[b]	9.52±0.85[a]	0.04
Clostridium_sensu_stricto_1	5.51±1.37	4.06±0.39	4.70±0.88	0.80
Bacteroides	0.90±0.16[a]	0.88±0.12[a]	0.37±0.04[b]	<0.01
Streptococcus	0.98±0.58	0.40±0.03	0.53±0.11	0.92
Aeromonas	0.01±0.00	0.01±0.00	0.18±0.17	0.13
Methanobrevibacter	3.49±0.88	1.55±0.38	1.73±0.45	0.08
Comamonas	0.18±0.09	0.17±0.08	0.04±0.02	0.15
Limosilactobacillus	0.13±0.03[b]	0.68±0.23[a]	0.71±0.13[a]	0.01
Others	69.67±2.07	74.09±2.42	73.12±1.49	0.28
Lactating sows				
Lactobacillus	4.02±0.86	1.50±0.44	2.42±0.45	0.06
UCG-002	9.34±1.72	8.10±0.93	7.51±1.09	0.61
Treponema	4.90±0.38	4.86±1.36	6.36±1.43	0.58
Christensenellaceae_R-7_group	3.87±0.36	5.26±0.97	5.04±1.47	0.54
Methanobrevibacter	1.42±0.26	0.98±0.27	1.80±0.09	0.05
Pseudomonas	0.53±0.11	3.51±1.87	0.53±0.14	0.55
Prevotella	2.27±0.16[a]	1.76±0.32[ab]	1.29±0.19[b]	0.02
Limosilactobacillus	1.47±0.29	0.50±0.19	0.97±0.25	0.07
Rikenellaceae_RC9_gut_group	5.07±0.59	4.07±0.36	4.61±0.71	0.55
Bacteroides	0.81±0.13	0.50±0.13	0.66±0.19	0.40
Others	64.65±2.26	64.73±0.90	66.72±2.18	0.72
Piglets				
Lactobacillus	4.99±0.86	4.16±0.98	7.82±2.95	0.84
Bacteroides	4.59±1.08	14.66±4.38	11.34±2.95	0.08
UCG-002	4.76±1.38	1.92±0.34	2.21±0.63	0.07
Fusobacterium	0.73±0.24[a]	2.64±1.08[b]	0.10±0.04[a]	0.01
Escherichia-Shigella	5.65±1.45	7.86±0.71	7.01±2.40	0.67
Rikenellaceae_RC9_gut_group	0.35±0.13	0.86±0.54	1.20±0.34	0.09
Limosilactobacillus	4.75±1.39	2.07±0.36	4.28±0.99	0.21
Parabacteroides	1.89±0.21[b]	2.22±0.41[b]	6.91±1.78[a]	0.01
Prevotella	0.77±0.23	0.87±0.27	0.15±0.02	0.05
Others	67.15±3.39[a]	50.75±4.52[b]	49.70±3.07[b]	<0.01

Data are expressed as mean ± standard error. CON group: basal diet; 0.125% group: basal diet + 1.25g /kg Y-dP; 0.2% group: basal diet +2 g/kg Y-dP. [a, b] Different superscript lowercase letters indicate significant differences ($P < 0.05$). n=8.
